# Supplementary material for: Bombyx mori Ecdysone Receptor B1 May Inhibit BmNPV Infection by Triggering Apoptosis
Source: Insects. 2023 May 31;14(6):505. doi: 10.3390/insects14060505 (PMC10299522; doi:10.3390/insects14060505)
Supplement: Supplementary file 1 [file insects-14-00505-s001.zip › insects-2337332-supplementary.pdf]

Supplementary Materials

Bombyx mori Ecdysone Receptor B1 May Inhibit BmNPV Infection by Triggering Apoptosis

Table S1. The list of accession numbers of BmEcR-B1 homologous in other species.

Species	Accession No.
Bombyx mandarina	XP_028037877.1
Galleria mellonella	XP_026757745.1
Trichoplusia ni	XP_026744328.1
Manduca sexta	XP_030038451.1
Spodoptera frugiperda	XP_035436715.1
Helicoverpa zea	XP_047021243.1
Spodoptera exigua	ACA30302.1
Helicoverpa armigera	XP_021181319.1
Thalassodes immissaria	UNA06120.1
Spodoptera littoralis	ADO64595.1
Spodoptera litura	AFK27930.1
Adoxophyes honmai	BBD75650.1
Antheraea pernyi	ARD05165.1
Ectropis obliqua	AKC43542.1

Table S2. The list of accession numbers of BmEcR-A homologous in other species

Species	Accession No.
Manduca sexta	XP_030038436.1
Spodoptera exigua	ADK66917.1
Omphisa fuscidentalis	ABS00248.1
Colias croceus	XP_045491669.1
Helicoverpa armigera	XP_049695177.1
Pieris rapae	XP_022112668.1
Pieris napi	XP_047512054.1
Pieris brassicae	XP_045532683.1
Zerene cesonia	XP_038215878.1
Hyposmocoma kahamanoa	XP_026330046.1
Spodoptera frugiperda	XP_035436717.1
Colias croceus	XP_045491666.1
Ostrinia furnacalis	XP_028170773.1
Spodoptera litura	XP_022819531.1
Choristoneura fumiferana	AAC61596.2:1
Vanessa cardui	XP_046964987.1
Nymphalis io	XP_050345605.1
Maniola jurtina	XP_045785074.1
Chilo suppressalis	BAC11713.1
Papilio machaon	XP_014370464.1
Pectinophora gossypiella	XP_049867465.1
Maniola hyperantus	XP_034840655.1

Danaus plexippus	XP_032513501.1
Plutella xylostella	XP_037973369.1
Leguminivora glycinivorella	XP_047988112.1
Conopomorpha sinensis	AQQ79920.1
Aricia agestis	XP_041977718.1
Plutella xylostella	XP_037973367.1
Anticarsia gemmatalis	UBY12696.1
Spodoptera litura	AFC87968.1

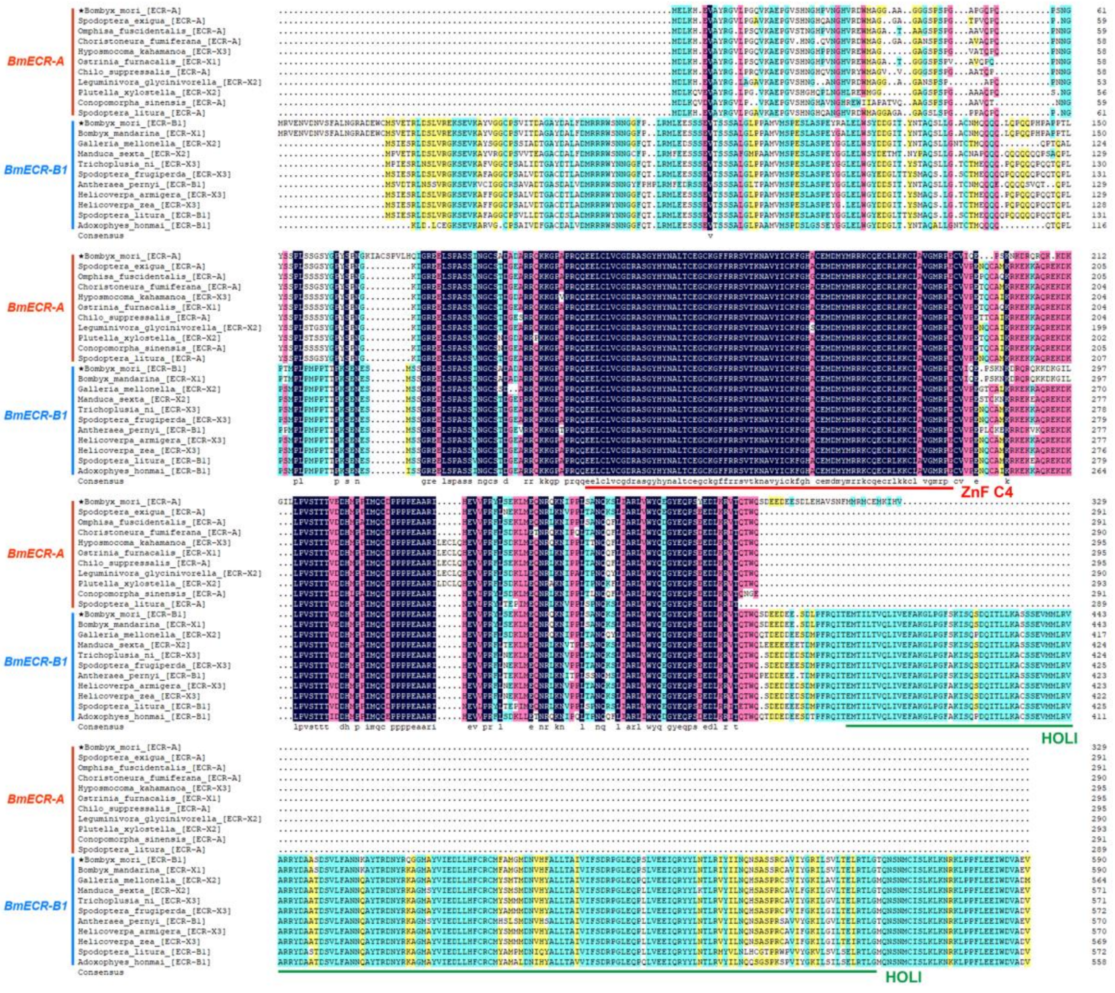

Figure S1. homologous alignment of the EcR-A and EcR-B1 amino acid sequences in different species. The amino acid sequence of the ZnF C4 domain is conserved in BmEcR-A and BmEcR-B1, while the HOLI domain is absent in BmEcR-A. The conserved amino acid sequence is highlighted in navy blue. The differential sequence is highlighted in pink, yellow, and light blue. The red underline denotes the ZnF C4 domain, and the green underline denotes the HOLI domain. ★, *Bombyx mori*.

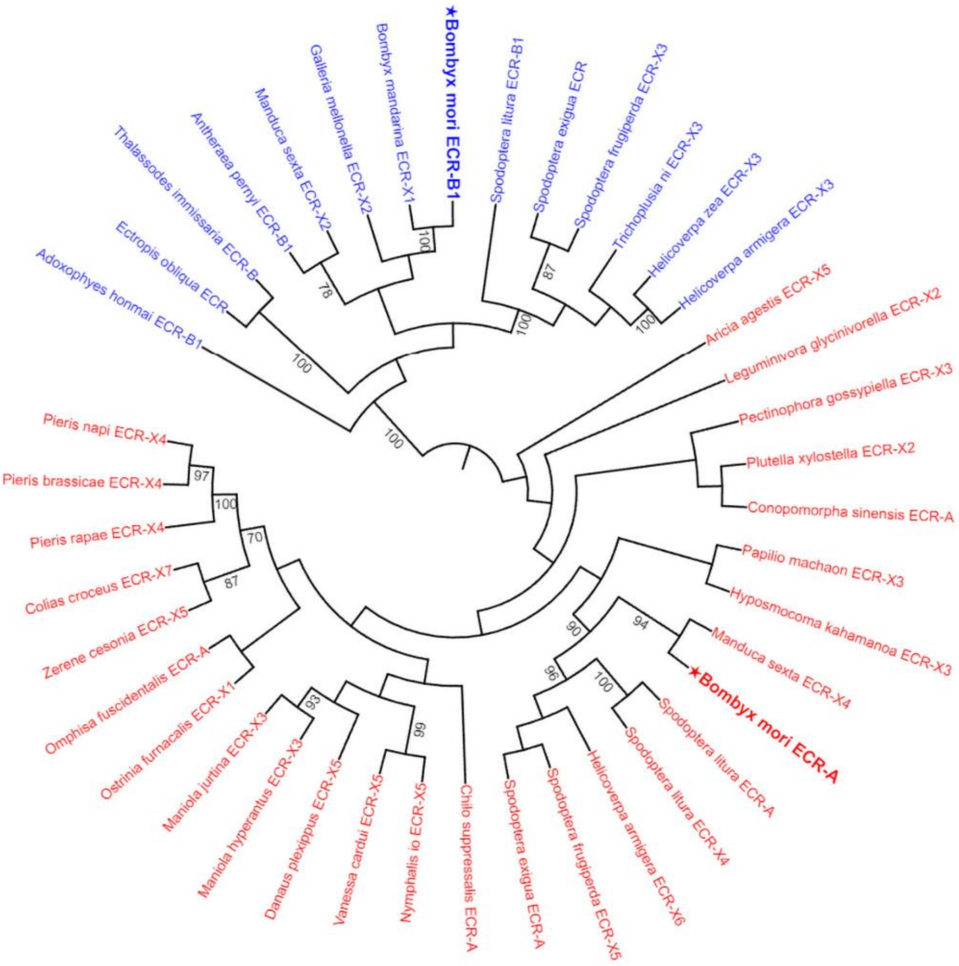

Figure S2. the phylogenetic tree of EcR-A and EcR-B1 in different species. The evolutionary relationship of EcR-A and EcR-B1 in different species was clustered into two separate groups, suggesting the function of BmEcR-B1 might be different from that of BmEcR-A. The star denotes EcR-A and EcR-B1 in Bombyx mori. BmEcR-B1 and its homologs are in blue, and BmEcR-A and its homologs are in red.

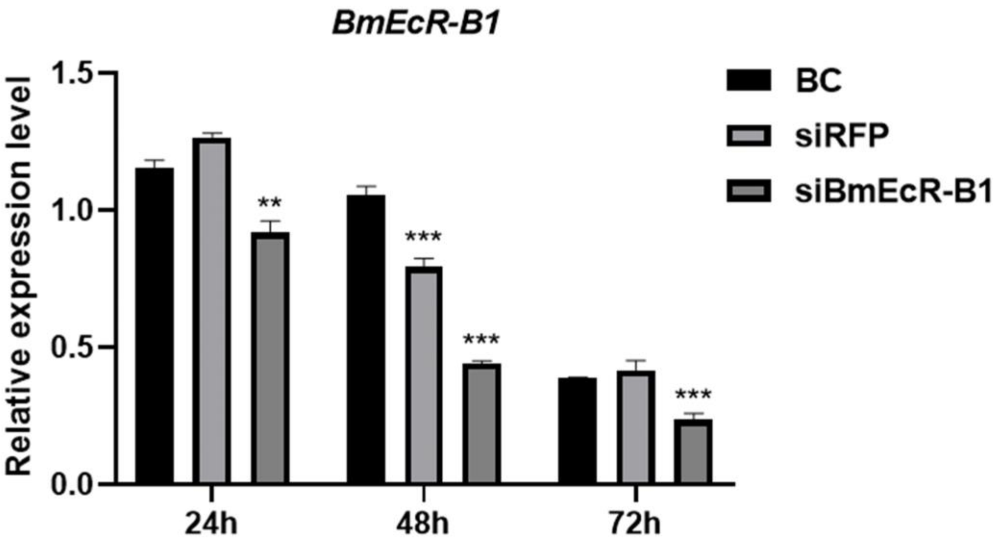

Figure S3. analysis of *BmEcR-B1* expression in BmN cells following 24 h, 48 h, and 72 h of siEcR-B1 transfection. .
